# Potential of Newborn and Adult Stem Cells for the Production of Vascular Constructs Using the Living Tissue Sheet Approach

**DOI:** 10.1155/2015/168294

**Published:** 2015-10-04

**Authors:** Jean-Michel Bourget, Robert Gauvin, David Duchesneau, Murielle Remy, François A. Auger, Lucie Germain

**Affiliations:** ^1^Université Laval Experimental Organogenesis Center/LOEX, Enfant-Jesus Hospital, 1401 18th rue, Québec, QC, Canada G1J 1Z4; ^2^Regenerative Medicine Section, CHU de Québec Research Centre, Québec, QC, Canada G1J 1Z4; ^3^Department of Surgery, Faculty of Medicine, Université Laval, Québec, QC, Canada G1V 0A6; ^4^Maisonneuve-Rosemont Hospital Research Center, 415 Assomption boulevard, Montreal, QC, Canada H1T 2M4; ^5^Department of Ophthalmology, University of Montreal, Montreal, QC, Canada H3T 1J4; ^6^Quebec Center for Functional Materials (CQMF), Office 2634, Alexandre-Vachon Building, Université Laval, Québec, QC, Canada G1V 0A6; ^7^Bordeaux Segalen University, INSERM-U1026, 146 Léo Saignat Street, 33000 Bordeaux, France

## Abstract

Bypass surgeries using native vessels rely on the availability of autologous veins and arteries. An alternative to those vessels could be tissue-engineered vascular constructs made by self-organized tissue sheets. This paper intends to evaluate the potential use of mesenchymal stem cells (MSCs) isolated from two different sources: (1) bone marrow-derived MSCs and (2) umbilical cord blood-derived MSCs. When cultured* in vitro*, a proportion of those cells differentiated into smooth muscle cell- (SMC-) like cells and expressed contraction associated proteins. Moreover, these cells assembled into manipulable tissue sheets when cultured in presence of ascorbic acid. Tubular vessels were then produced by rolling those tissue sheets on a mandrel. The architecture, contractility, and mechanical resistance of reconstructed vessels were compared with tissue-engineered media and adventitia produced from SMCs and dermal fibroblasts, respectively. Histology revealed a collagenous extracellular matrix and the contractile responses measured for these vessels were stronger than dermal fibroblasts derived constructs although weaker than SMCs-derived constructs. The burst pressure of bone marrow-derived vessels was higher than SMCs-derived ones. These results reinforce the versatility of the self-organization approach since they demonstrate that it is possible to recapitulate a contractile media layer from MSCs without the need of exogenous scaffolding material.

## 1. Introduction

Cardiovascular diseases (CVD) are the leading cause of mortality in the United States (US) [1]. Half of the CVD-associated fatalities are attributed to myocardial infarct caused by obstruction of a coronary artery [1]. The gold standard for vascular bypass surgeries of small diameter/low blood flow vessels is the transplantation of an autologous vein or artery [2]. However, these vessels are limited in availability and could be pathologic [3–5]. Therefore, there is an urgent need for the development of an alternative conduit that could be nonthrombogenic and nonimmunogenic and present proper mechanical properties as well as vasoreactivity [6, 7]. Such vessels can be engineered from autologous cells using the self-organization approach, which takes advantage of the capability of mesenchymal cells to produce and assemble their own extracellular matrix (ECM) when cultured in presence of ascorbic acid [8, 9]. In a seminal paper by L'Heureux et al. [9] the adventitia was recapitulated using adult dermal fibroblasts (DFs) while the media layer was engineered from umbilical vein smooth muscle cells (SMCs) and the intima from umbilical vein endothelial cells. However, this source of SMCs is not compatible with a fully autologous approach. In order to fulfill this expectancy, it would be preferable to have access to another potential source of SMCs.

Previous studies have focused on the evaluation of alternative cell sources for media reconstruction by the self-organization approach (formerly called the self-assembly approach). Grenier et al. [10] developed a protocol for the isolation of the 3 vascular cell types (endothelial, SMC, and fibroblast) from a single vein biopsy. This interesting approach faces, however, the potential problems, for some patients, of the cells taken from a pathologic vessel such as the saphenous vein. Moreover, it is an invasive approach since it requires surgery and an available vein, which can be difficult to access for patients with history of multiple autologous vascular bypass grafts. Additionally, tissue-engineered vessels produced from cells isolated from veins have demonstrated inferior mechanical properties than those reconstructed from arterial cells [11].

The use of adult mesenchymal stem cells (MSCs) could overcome the challenge of using cells from pathologic tissues. MSCs are known to be able to differentiate into multiple mesenchymal cell types (cardiomyocytes, osteocytes, chondrocytes, myocytes, fibroblasts, and adipocytes) [12]. They can also spontaneously differentiate into smooth muscle cells when cultured for several passages [13]. Moreover, those cells possess paracrine effects such as immunomodulation, antiapoptosis, antiscarring, and chemoattraction [12]. They can be obtained from different sources including adipose tissue, the umbilical cord, bone marrow, and the dermis [14–16]. The current theory on the MSCs niche is that these cells are located around capillaries and correspond to pericytes [17–19].

Previous work demonstrated the potential of those cells for tissue engineering applications using the self-organization approach. Hayward et al. have demonstrated that umbilical cord Wharton's jelly-derived MSCs can be used to reconstruct dermal and vascular constructs [20, 21]. Vermette et al. [22] and Rousseau et al. [23] have also used adipose tissue-derived stromal cell containing MSCs for engineered adipose tissue and bladder wall, respectively.

In order to find a suitable alternative source of cells, we studied the potential of bone marrow mesenchymal stem cells (BMSCs) and umbilical cord blood mesenchymal stem cells (UCB-MSCs) to differentiate in SMCs, assemble extracellular matrix, produce manipulable cell sheets, and form a cohesive vascular media substitute.

## 2. Materials and Methods

### 2.1. Cell Isolation and Culture

All protocols were approved by the institutional committee for the protection of human subjects (Comité d'Éthique de la Recherche du Centre Hospitalier Universitaire de Québec) and conducted in accordance with the Declaration of Helsinki.

Umbilical cord blood mesenchymal stem cells (UCB-MSCs) were isolated from the mononuclear fraction of samples of freshly collected human umbilical cord blood samples (50–80 mL), using an adaptation of the previously described protocol by Kaushal et al. [24]. Briefly, after elimination of the plasma and platelets by centrifugation, the erythrocytes were sedimented in 6% dextran (Sigma-Aldrich, Oakville, Ontario, Canada). The mononuclear fraction was then isolated by centrifugation through a Ficoll-Paque gradient (Amersham Biosciences, Piscataway, NJ, USA) and resuspended in phosphate buffered saline (PBS). Cells were centrifuged and resuspended in M199 medium (Sigma-Aldrich) supplemented with 20% newborn calf serum (FetalClone II, HyClone, Logan, UT, USA), endothelial cell growth supplement (20 *μ*g/mL; Sigma-Aldrich), glutamine (333 *μ*g/mL; Life Technologies, Burlington, Ontario, Canada), heparin (40 U/mL; LEO Pharma Inc., Thornhill, Ontario, Canada), and antibiotics: 100 U/mL penicillin G (Sigma-Aldrich) and 25 g/mL of gentamicin (Schering, Pointe-Claire, QC, Canada). This mononuclear fraction obtained from cord blood was then plated in a Petri dish coated with 0.2% gelatin (Fisher Scientific, Ottawa, Ontario, Canada). Cells were allowed to adhere to the dish for 3 hours in M199 medium at 37°C to select the population of adherent cells. After 20 days, two distinct cell populations were visible in the flask, angioblastic-like cells and mesenchymal-like cells. These two populations were separated by differential trypsinization (trypsin; Life Technologies). The mesenchymal population was then cultured in Dulbecco's modified Eagle medium (DMEM, Life Technologies) and Ham's F12 (Life Technologies) in a 3 : 1 ratio (DMEM-Ham), 20% fetal calf serum (FCS; HyClone), and antibiotics.

Bone marrow mesenchymal stem cells (BMSCs) were bought from Lonza (Lonza, Walkersville, MD, USA) and cultured as prescribed by the manufacturer. This cell line has been characterized previously (CD105+, CD66+, CD29+, CD44+, CD34−, CD14−, and CD45−) and can be differentiated into adipogenic, chondrogenic, and osteogenic cells [25].

Human DFs were isolated from a healthy donor following breast surgery and cell isolation was performed as described previously [26]. Briefly, a small portion of skin was incubated at 37°C for 2 hours in a thermolysin (500 *μ*g/mL, Sigma-Aldrich)/HEPES buffer (pH 7.4, MP Biomedicals, Montreal, QC, Canada) solution. The dermis and epidermis were then separated using fine forceps. The dermis was cut into small pieces and incubated at 37°C for 20 hours in a collagenase H solution (0.125 U/mL, Roche, Laval, QC, Canada) in order to isolate fibroblasts from the connective tissue. Fibroblasts were centrifuged (300 ×g, 10 min), plated into culture flasks, and cultured in DMEM with 10% FCS and antibiotics.

Human arterial SMCs were isolated from an umbilical cord artery using the explants method of Ross [27, 28]. Briefly, the artery was longitudinally opened and the endothelium was removed by gentle scraping with a sterile gauze soaked in a PBS solution. Strips of the media layer were dissected, cut into small pieces, and allowed to adhere to a gelatin-coated 6-well plate (BD Bioscience, Mississauga, Ontario, Canada) containing culture medium: DMEM-Ham, 20% FCS, and antibiotics. After 2 weeks in culture, SMCs migrated out of the explants and were allowed to proliferate for two more weeks. SMCs were detached from the tissue culture plastic using trypsin/EDTA and further plated at a density of 10^4^ cells/cm^2^ in tissue culture flasks to allow for expansion.

All cell types were grown in an incubator at 8% CO_2_, 95% relative humidity (RH), and 37°C and the culture medium was changed 3 times a week.

### 2.2. Production of Tissue-Engineered Vascular Constructs

The tissue-engineered vascular constructs were produced using the self-organization technique previously described [9, 29]. Briefly, cells were seeded on tissue culture flask (T75, BD Bioscience) in DME-Ham (3 : 1), supplemented with 10% FCS and 50 *μ*g/mL ascorbic acid. After 3 weeks in culture, cell sheets were peeled off the flask and rolled around mandrels (4.5 mm diameter) to generate a vascular construct. These were cultured for an additional three weeks in DMEM-Ham supplemented with 10% FetalClone II serum, antibiotics, and 50 *μ*g/mL of sodium ascorbate.

### 2.3. Immunostaining

For immunostaining on coverslips, cells were fixed in 100% methanol (−20°C, 10 min) and rinsed in PBS. For immunostaining of cross sections, segments of tissue-engineered vessels were embedded in optimal cutting temperature compound (OCT, Tissue-Tek/Somagen, Edmonton, Alberta, Canada) and frozen at −80°C. OCT embedded tissues were cut orthogonal to the length of the vascular construct in 5 *μ*m sections using a cryostat (Leica Canada, Montreal, QC, Canada), fixed in 100% acetone (−20°C, 10 min), and rinsed in PBS. Primary antibodies used were mouse monoclonal antibodies (IgG) against *α*-smooth muscle actin (clone 1A4; Dako, Burlington, Ontario, Canada; 1/200), calponin (clone hCP; Sigma-Aldrich; 1/200), h-caldesmon (clone hHCD; Sigma-Aldrich; 1/100), and keratin-18 (clone KS18.174; ARP, Waltham, MA, USA; 1/100). Secondary antibody was goat polyclonal antibody against mouse IgG conjugated with Alexa 594 (Life Technologies; 1/800).

### 2.4. Histology

Segments of constructs were fixed in 3,7% formaldehyde (VWR, Montreal, QC, Canada) and embedded in paraffin. Five-micrometer thick cross sections were cut using a microtome and stained with Masson's trichrome [30, 31] using Weigert's haematoxylin, fuchsin-ponceau, and aniline blue stains.

### 2.5. Burst Pressure Test

Burst pressure measurements were performed by gradually inflating the tissue-engineered constructs until failure, while recording the internal pressure, using a custom-built experimental setup described previously [32]. Briefly, tissues were cannulated and loaded in a PBS containing chamber maintained at 37°C. Pressurization of vascular constructs with PBS was ensured by a syringe pump activated by a stepper motor (Excitron, Boulder, CO, USA) and controlled by a LabView virtual instrument (National Instruments, Austin, TX), at a constant flow rate of 4 mL/min. Pressure data were recorded by a pressure transducer (68846-series; Cole Parmer, Montreal, QC, Canada) connected to an acquisition card (NI PCI-6221; National Instruments) and acquired using the previously described virtual instrument [32]. Burst pressure was considered to be the highest pressure value recorded prior to failure of the construct.

### 2.6. Uniaxial Tensile Test

Mechanical properties of the vascular constructs were evaluated by ring tensile testing using an Tytron 250 Microforce Testing System (MTS Corporation, Eden Prairies, MN, USA) as previously described [32, 33]. Briefly, five-millimetre-long ring samples were cut from the different constructs and mounted between hooks linked to a 10 N load cell. The hooks were pulled apart at the constant speed of 0.2 mm/s until failure of the specimen.

### 2.7. Vasoconstriction

The contractile properties of the tissue-engineered vessels were evaluated by recording their response to histamine (Sigma-Aldrich) as previously described [34, 35]. Briefly, the different constructs were cut into five-millimetre-long ring sections, rinsed in physiological salt Krebs solution (119 mM NaCl, 4.7 mM KCl, 1.2 mM KH_2_PO_4_, 25 mM NaHCO_3_, 1.2 mM MgSO_4_, 2.5 mM CaCl_2_, and 10 mM glucose), mounted between two anchors for isometric contractile force measurements (Radnoti, Harvard Apparatus, Montreal, QC, Canada), and submerged in isolated organ baths containing Krebs solution maintained at 37°C and oxygenated with a mixture of 95% O_2_ and 5% CO_2_ (pH 7.4). After 30 minutes of equilibration, each ring was passively stretched until a stable preload of 500 mg was obtained. The maximal contractile capability of each ring was determined by a single dose of histamine (10^−4^ M).

## 3. Results

### 3.1. Cultured MSCs Express SMC Differentiation Markers* In Vitro*

**Figure 1:**
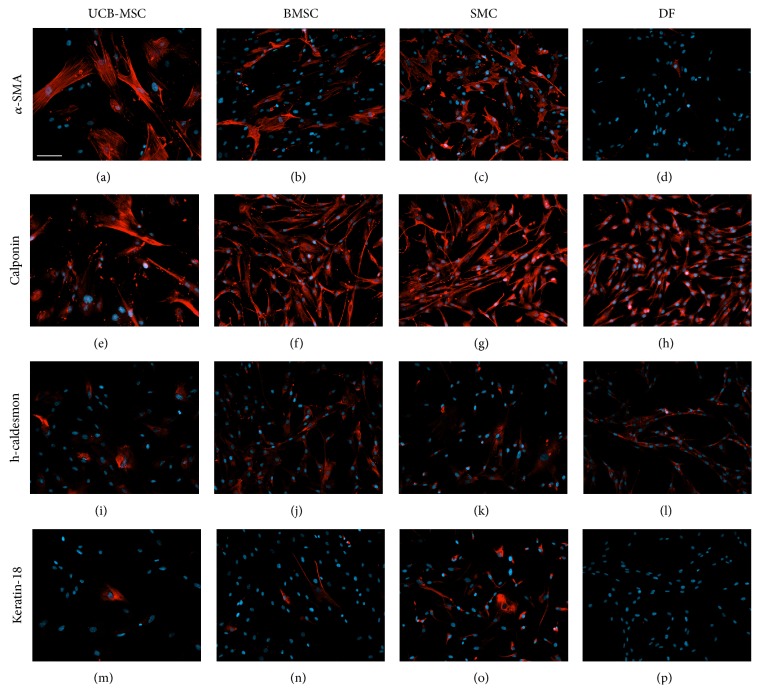
Cultured MSCs express smooth muscle cell markers. Expression of smooth muscle cell markers by various cell types in culture, UCB-MSCs (a, e, i, and m) and BMSCs (b, f, j, and n), as well as control cells, SMCs (c, g, k, and o) and DFs (d, h, l, and p). Immunostaining against (red) *α*-smooth muscle (SM) actin (a–d), calponin (e–h), h-caldesmon (i–l), and keratin-18 (m–p). Nuclei were counterstained with Hoechst (blue); scale bar: 100 *μ*m.

In order to evaluate the extent of the differentiation of UCB-MSCs and BMSCs into SMCs, cells were characterized for expression of SMC markers by immunofluorescence at different passages and compared to SMCs and DFs (Figure 1). At passage 10, both types of MSCs were positive for the expression of *α*-smooth muscle actin (*α*-SMA) (Figures 1(a)–1(d)) and calponin (Figures 1(e)–1(h)), two early SMC markers. Similarly, DFs also stained positive for calponin which can be explained by a transition of those cells toward a myofibroblastic phenotype in culture. Expression of h-caldesmon (Figures 1(i)–1(l)), a later stage SMC marker, was also tested. This protein was expressed by a small proportion of UCB-MSCs (30% of cells) but was present in each cell population tested. Expression of h-caldesmon was high in BMSCs (100% of cells) (Figure 1(j)). Most SMCs stained positive for this marker although some of them did not express h-caldesmon (Figure 1(k)), since SMCs are known to dedifferentiate from their contractile phenotype to a proliferative phenotype when cultured* in vitro* [36]. Keratin-18 is known to be expressed by SMCs but not by dermal fibroblasts [37–39]. This protein was expressed by a small proportion of UCB-MSCs (9%) and BMSCs (14%) but by a high proportion of SMCs (87%). As expected, DFs were negative for this marker.

### 3.2. MSCs Capability to Form Cell Sheets

**Figure 2:**
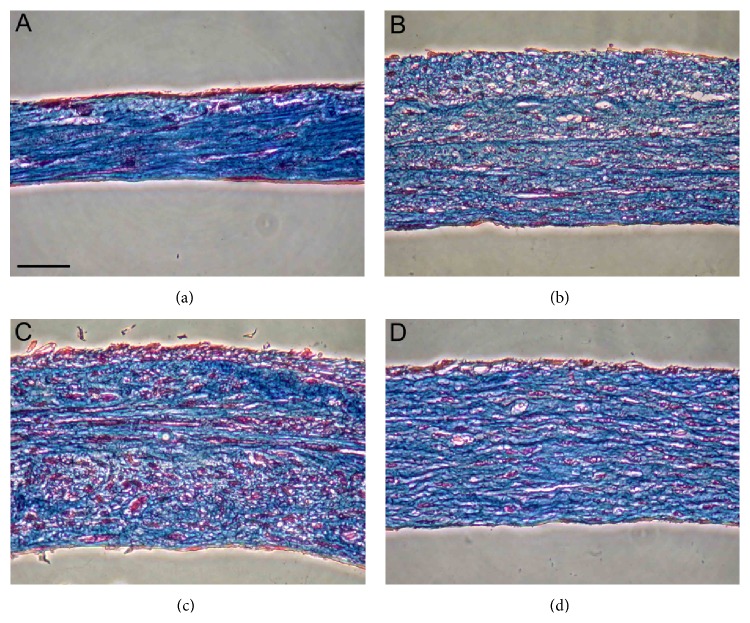
Cultured stem cells produce ECM and form cell sheets that can be later rolled into vascular constructs. Cross sections of tissue-engineered vessels made from cultured UCB-MSCs (a), BMSCs (b), SMCs (c), or DFs (d) were stained with Masson's trichrome in order to visualize collagen (blue) and cells (red). Scale bar: 50 *μ*m.

The BMSCs and UCB-MSCs were cultured in presence of ascorbic acid in order to evaluate their capability to secrete and assemble collagen using a previously described protocol [26]. Both types of MSCs secreted a sufficient amount of extracellular matrix to form cell sheets. However, UCB-MSCs formed fragile cell sheets that were hard to manipulate in comparison with their counterparts. MSCs-derived sheets were rolled around a mandrel to form vascular constructs. Those constructs and control ones (SMCs- and DFs-derived) were stained with Masson's trichrome to visualize cells and ECM (Figure 2). All cell types formed tubular constructs comprising cells embedded into a dense collagenous ECM. UCB-MSCs-derived vessels were much thinner than the others. This finding correlates with the previous observation of a thin and fragile cell sheet. However, all four types of constructs could be slit out from their support mandrel into culture medium and were able to maintain their internal lumen geometry without collapsing.

### 3.3. Vascular Constructs Produced from Stem Cells Express Contractile SMC Proteins

**Figure 3:**
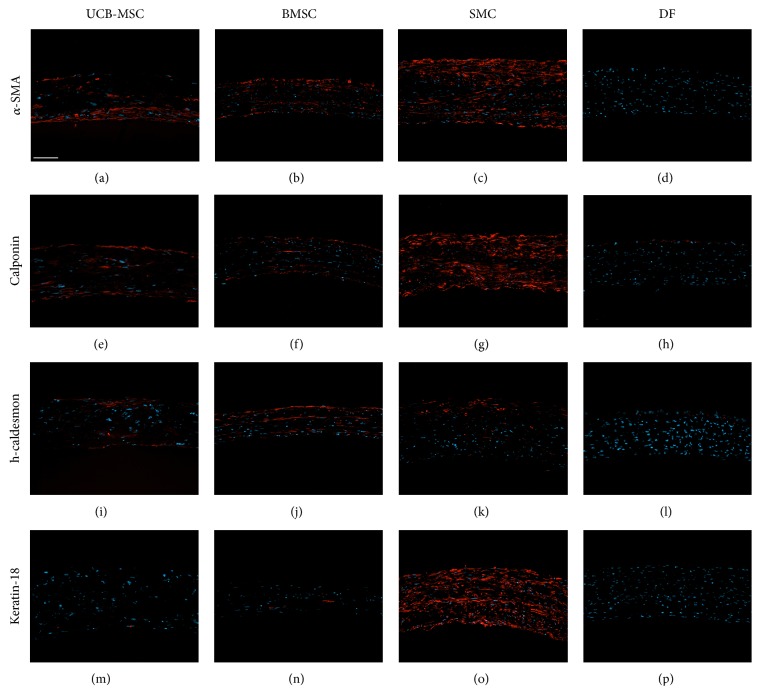
Expression of SMC differentiation markers in MSCs-derived vessels. Cross sections of tissue-engineered vessels made from cultured UCB-MSCs (a, e, i, and m), BMSCs (b, f, j, and n), SMCs (c, g, k, and o), or DFs (d, h, l, and p) were immunostained (red) for *α*-SMA (a–d), calponin (e–h), h-caldesmon (i–l), and keratin-18 (m–p). Nuclei were counterstained with Hoechst (blue); scale bar: 100 *μ*m.

In order to evaluate the expression of SMC markers in the vascular constructs derived from all four cells types, cross sections of tissue-engineered vessels were immunostained with the same markers as 2D cultures presented in Figure 1. SMCs-derived constructs stained positive for all four markers, namely, *α*-SMA, calponin, h-caldesmon, and keratin-18 (Figures 3(c), 3(g), 3(k), and 3(o)) while DFs-derived ones turned out to be negative for the same four markers (Figures 3(d), 3(h), 3(l), and 3(p)). MSCs-derived constructs, from UCB-MSCs (Figures 3(a), 3(e), 3(i), and 3(m)) and from BMSCs (Figures 3(b), 3(f), 3(j), and 3(n)), displayed an intermediate phenotype with a level of expression of SMC markers between the SMCs- and DFs-derived constructs.

### 3.4. Evaluation of the Contractile Capability of Stem Cell Derived Media

**Figure 4:**
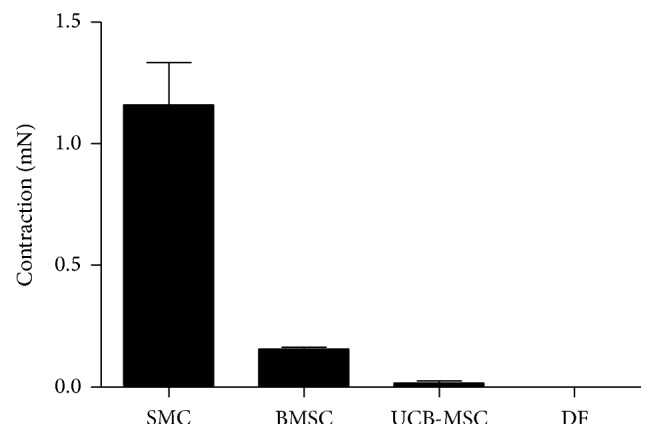
MSCs-derived media responsiveness to contractile agonist. Vasoreactivity of different tissue-engineered vessels. Recording of the maximal contraction to histamine (10^−4 ^M).

We have previously demonstrated that media produced by self-organization of tissue sheets from SMCs can contract in response to different agonist [34]. This contractile capability is paramount to proper function of this blood vessel layer. Being able to dilate or contract in response to local increases in pressure or blood flow might have significant impact on vascular functionality. Therefore, we evaluated the contractile response of the different engineered media to histamine. This molecule is known to induce the contraction of SMCs-derived constructs and has been previously validated by our group [35, 40–43]. The UCB-MSCs- and BMSCs-engineered vascular media produced a low level of contraction as compared to the SMCs-engineered media. The contraction of BMSCs-derived media was higher than UCB-MSCs-derived media. Still, the contraction monitored for those MSCs-derived media was higher than the contraction of the control, DFs-derived vessels (Figure 4). The agonist-dependent response of MSCs-derived media demonstrates that their contractile apparatus is functional and that they express the histamine receptor.

### 3.5. Evaluation of the Mechanical Properties of Stem Cell Derived Vascular Construct

**Figure 5:**
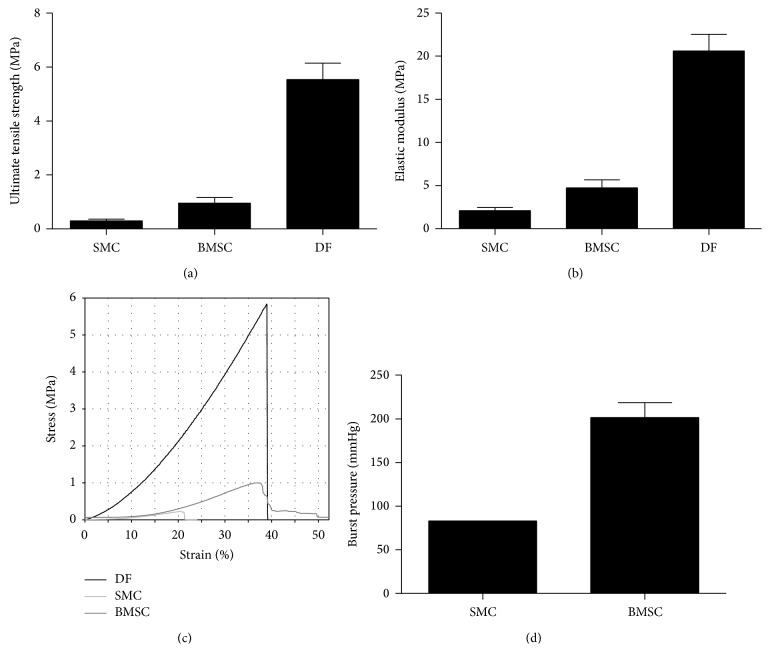
BMSCs-derived media present higher mechanical resistance than SMCs-derived media. Ultimate tensile stress (a) and elastic modulus (b) were determined by uniaxial tensile testing. A representative stress-strain curve is shown in (c). Burst pressure was evaluated on whole tissue-engineered vessels (d).

Suitable mechanical properties of a vascular substitute intended for transplantation are paramount. In order to evaluate those properties in the different constructs, tissues were subjected to two types of mechanical tests: uniaxial tensile tests (Figures 5(a), 5(b), and 5(c)) and burst pressure tests (Figure 5(d)). Mechanical resistance of UCB-MSCs-derived constructs was too low to be determined accurately. Results have shown that BMSCs could lead to vascular construct displaying mechanical properties within the same order of magnitude when compared to SMCs and DFs.

## 4. Discussion

We have shown that it is possible to engineer a contractile media layer from adult and newborn MSCs. Those constructs expressed SMCs differentiation markers and formed a cohesive tubular construct. Adult MSCs isolated from the bone marrow presented superior properties over UCB-MSCs since their contractile capability was found to be closer to SMCs-derived constructs and they present a higher mechanical resistance than UCB-MSCs.

Nowadays, use of MSCs in tissue engineering and regenerative medicine is quite common [44–49]. These cells have been used for vascular tissue engineering [50] in scaffold based approaches such as ECM-based scaffold (fibrin) [51], decellularized tissue [52, 53], and biodegradable scaffolds [54–56], including electrospun of nanofibers [57–59], as well as scaffold-free approach such as cell sheet engineering [60] and the present paper.

Ren et al. [61] have shown that MSCs cell sheets seeded with endothelial cells promote the formation of a microvascular network in the construct. This is quite interesting since the formation of a vasa vasorum in the vascular adventitia of tissue-engineered vessels [62] and tissue-engineered skin [63], using dermal fibroblasts, improves the graft integration and inosculation.

There are advantages of using mesenchymal stem cells for reconstruction of the vascular media. Mechanical resistance of BMSCs construct was found to be higher than SMCs-derived constructs. The BMSCs are readily available from a simple bone marrow biopsy and can be expanded* in vitro*. They are also available commercially for research purposes. They form cell sheets that can improve capillary formation. However, there is also inherent drawback to take into account. Contractile response to vasocontractile agonist is lower than SMCs-derived constructs. Interestingly, contraction intensity seems to correlate with the expression of SMC contractile apparatus proteins. Indeed, more cells expressed h-caldesmon in BMSCs culture than UCB-MSCs. Accordingly, BMSCs-derived media present a higher contractile capability. It is also likely that mechanical stimulation of the construct might be able to improve the contractile response after grafting [33]. This mechanical stimulation could also be simulated* in vitro* in a bioreactor. Indeed, previous studies have shown that applying cyclic strain to SMC tissue sheets increased mechanical resistance and contractility [64]. Uniaxial mechanical stimulation of DF tissue sheets also increased ultimate tensile strength [65]. The same phenomenon could probably be observed for tissue sheets engineered using BMSCs, since cell type is known to be influenced by mechanical stimulation [66, 67].

## 5. Conclusion

This study demonstrated the feasibility of producing a contractile media layer* in vitro* from adult and newborn MSCs using the self-organization approach. The cohesive tubular construct contained cells expressing SMCs differentiation markers. Adult BMSCs were found to be preferable to replace SMCs isolated from the vessels, compared to UCB-MSCs, to reconstruct a media layer. Contractile capability of BMSCs was closer to SMCs-derived constructs and they presented a higher mechanical resistance when compared to UCB-MSCs. Those cells could potentially be isolated from the patients' bone marrow in an autologous approach. The use of MSCs in tissue engineering might be the key autologous reconstruction of blood vessels, especially for patients lacking available healthy tissue for bypass surgeries.
